# MDR and Pre-XDR Clinical *Mycobacterium tuberculosis* Beijing Strains: Assessment of Virulence and Host Cytokine Response in Mice Infectious Model

**DOI:** 10.3390/microorganisms9081792

**Published:** 2021-08-23

**Authors:** Mikhail V. Fursov, Egor A. Shitikov, Denis A. Lagutkin, Anastasiia D. Fursova, Elena A. Ganina, Tatiana I. Kombarova, Natalia S. Grishenko, Tatiana I. Rudnitskaya, Dmitry A. Bespiatykh, Nadezhda V. Kolupaeva, Viktoria V. Firstova, Lubov V. Domotenko, Anna E. Panova, Anatoliy S. Vinokurov, Vladimir A. Gushchin, Artem P. Tkachuk, Irina A. Vasilyeva, Vasiliy D. Potapov, Ivan A. Dyatlov

**Affiliations:** 1 State Research Center for Applied Microbiology and Biotechnology, Territory “Kvartal A”, 142279 Serpukhov, Russia; anfursova06@gmail.com (A.D.F.); ganin43@yandex.ru (E.A.G.); kombarova.tatyana@yandex.ru (T.I.K.); natalya.grishenko60@mail.ru (N.S.G.); rudnitskaya.tat@yandex.ru (T.I.R.); nadin.9830@mail.ru (N.V.K.); firstova@obolensk.org (V.V.F.); domotenko@obolensk.org (L.V.D.); potapovvd@mail.ru (V.D.P.); dyatlov@obolensk.org (I.A.D.); 2 Federal Research and Clinical Center of Physical-Chemical Medicine, 119435 Moscow, Russia; eshitikov@mail.ru (E.A.S.); d.bespiatykh@gmail.com (D.A.B.); 3 National Medical Research Center for Phthisiopulmonology and Infectious Diseases of the Ministry of Health of the Russian Federation, 127473 Moscow, Russia; lagutkin.da@phystech.edu (D.A.L.); panovaae@nmrc.ru (A.E.P.); anatol-vinok@mail.ru (A.S.V.); director@nmrc.ru (I.A.V.); 4 N.F. Gamaleya National Research Centre for Epidemiology and Microbiology of the Ministry of Health of the Russian Federation, 123098 Moscow, Russia; wowaniada@gmail.com (V.A.G.); artem.p.tkachuk@gmail.com (A.P.T.)

**Keywords:** *Mycobacterium tuberculosis*, Beijing, tuberculosis (TB), whole-genome sequencing (WGS), resistance, infectious diseases, mice model, cytokines

## Abstract

*Mycobacterium tuberculosis* Beijing genotype associated with drug resistance is a growing public health problem worldwide. The aim of this study was the assessment of virulence for C57BL/6 mice after infection by clinical *M. tuberculosis* strains 267/47 and 120/26, which belong to the modern sublineages B0/W148 and Central Asia outbreak of the Beijing genotype, respectively. The sublineages were identified by the analysis of the strains’ whole-genomes. The strains 267/47 and 120/26 were characterized as agents of pre-extensively drug-resistant (pre-XDR) and multidrug-resistant (MDR) tuberculosis, respectively. Both clinical strains were slow-growing in 7H9 broth compared to the *M. tuberculosis* H37Rv strain. The survival rates of C57BL/6 mice infected by 267/47, 120/26, and H37Rv on the 150th day postinfection were 10%, 40%, and 70%, respectively. Mycobacterial load in the lungs, spleen, and liver was higher and histopathological changes were more expressed for mice infected by the 267/47 strain compared to those infected by the 120/26 and H37Rv strains. The cytokine response in the lungs of C57BL/6 mice after infection with the 267/47, 120/26, and H37Rv strains was different. Notably, proinflammatory cytokine genes *Il-*1*α*, *Il-*6, *Il-*7, and *Il-*17, as well as anti-inflammatory genes *Il-*6 and *Il-*13, were downregulated after an infection caused by the 267/47 strain compared to those after infection with the H37Rv strain.

## 1. Introduction

Tuberculosis (TB), caused by *Mycobacterium tuberculosis* complex bacteria, is one of the biggest public health problems worldwide, especially in developing countries. In 2019, about 10 million people developed TB and 1.4 million died of it [1]. Currently, *M. tuberculosis* is classified into eight lineages, of which lineage 2, with its main Beijing genotype, is a major component of the global pathogen population [2,3]. An analysis of a large dataset showed that Beijing strains are distributed worldwide and are associated both with treatment failure in some countries and with an increased risk of transmission chain formation [4,5]. This may owe to the ability to possess selective advantages in comparison to strains from other mycobacterial lineages, increased capacity to acquire drug resistance and compensatory mutations, and/or more rapid progression to disease after infection [6,7].

The listed features of the Beijing genotype are undoubtedly associated with the genetic background of the bacterium. However, genetic diversity within genotype members leaves an imprint on itself: data on the virulence assessment of Beijing isolates are inconclusive, showing a broad range of inflammatory and virulence phenotypes, as determined in animal and in vitro models of macrophage infection [8,9,10].

Today, lineage 2 comprises at least 11 sublineages belonging to two main branches—ancient and modern Beijing [11]. Commonly, modern Beijing strains demonstrate greater pathogenicity than the ancient, which agrees with the conclusions of genetic and epidemiological studies [12,13]. However, the correlation between sublineage and virulent properties is not unambiguous for all strains of the Beijing genotype. Differences can be found even for the most studied clusters, Beijing B0/W148 and Central Asia outbreak (CAO). Members of these clusters are regarded as “successful” clones of the genotype and are widespread in Russia and some countries of the former Soviet Union [14,15,16].

Beijing B0/W148 strains demonstrated increased virulence in experiments in mice macrophage models compared to other variants of the Beijing genotype [17]. Similar results were demonstrated in the monocyte-like cell line THP-1 [18]. At the same time, experiments using animal models yielded different results [12,19]. Furthermore, Beijing CAO strains were also shown to be associated with different levels of virulence for mice. It was reported that a strain of the CAO sublineage showed the same level of virulence for C57BL/6 mice as reference strain H37Rv [20]. In contrast, we recently characterized the strain Rostov, which was significantly more virulent for C57BL/6 mice than the strain H37Rv, as belonging to the CAO sublineage [21].

One of the pathogenic factors of *M. tuberculosis* strains is their ability to evade a host’s immune response. In recent years, the cytokine induction at infection caused by *M. tuberculosis* strains from different genetic lineages has been estimated in several studies. It was hypothesized that the lower levels of induction of proinflammatory cytokines by modern Beijing strains may help them to spread across the globe [22]. Beijing B0/W148 strains induced reduction over time of TNF secretion and a high level of IL-10 production following immunosuppression compared with the H37Rv strain [18]. The CAO strains have not yet been studied in this direction.

In the present study, we estimated the virulence of two clinical Beijing B0/W148 and CAO *M. tuberculosis* strains on C57BL/6 mice in comparison with that of the *M. tuberculosis* H37Rv reference strain. The following criteria were used to assess the virulence of the strain: mice survival rate and bodyweight dynamic, mycobacterial load, histological analysis of mice parenchymal organs, and assessment of cytokine gene transcription in the lung. Moreover, microbiological and genomic characteristics of the strains were obtained such as the growth index, C_max_, and antibacterial susceptibility; drug resistance markers and compensatory mutations were identified; and phylogenetic analysis was conducted versus 506 Beijing B0/W148 and 183 CAO isolates retrieved from the GenBank database. This is an extensive study of the virulence properties of the clinical B0/W148 strain and the CAO strain, including genome analysis and cytokine gene transcription analysis.

## 2. Materials and Methods

### 2.1. Bioethical Requirements

The materials used in the study did not contain the personal data of patients because the labeling of the clinical isolates did not include name, date of birth, address, or other personal information. Under the requirements of the Russian Federation Bioethical Committee, each patient signed an agreement with the hospital consenting to treatment and laboratory examination. All animal experiments were carried out according to the requirements of the European Convention for the Protection of Vertebrate Animals Used for Experimental and Other Scientific Purposes (Directive 2010/63/EU of the European Parliament and of the Council of 22 September 2010 on the protection of animals used for scientific purposes), Sanitary Regulation 1.3.2322-08 “Safety of work with microorganisms of the III-IV pathogenicity groups and pathogens of parasitic diseases”, and Veterinary Protocol No. VP-2020/10. In addition, all animal experiments were approved by the bioethics division of the State Research Center for Applied Microbiology and Biotechnology.

### 2.2. Sputum Collection and Processing

Sputum samples were collected under standard TB program conditions from patients being tested for TB. Specimens were cooled at 2–8 °C until transportation and decontaminated with a BBL MycoPrep System (BD, Franklin Lakes, NJ, USA). Processed samples were concentrated with centrifugation at 3000× *g* and 4 °C for 15 min. Zero point five milliliters of decontaminated concentrated specimens were inoculated to Löwenstein–Jensen medium (BD, Franklin Lakes, NJ, USA). Samples 264/47 and 120/26 were then randomly selected from the culture collection in NMRC of Phthisiopulmonology and Infectious Diseases.

### 2.3. Strains and Growth Conditions

*M. tuberculosis* strains 267/47 and 120/26 were isolated from the sputum of two men hospitalized in the National Medical Research Center for Phthisiopulmonology and Infectious Diseases of the Ministry of Health of the Russian Federation on 18 April 2018 and 18 May 2018, respectively. The clinical strains were deposited into the State Collection of Pathogenic Microorganisms (SCPM–Obolensk) as ID B-9344 and B-9343, respectively. The virulent laboratory strain *M. tuberculosis* H37Rv was obtained from the SCPM–Obolensk (ID B-4825). Mycobacterial cells were grown in Middlebrook 7H9 broth with OADC supplement (BD, Franklin Lakes, NJ, USA) containing 0.05% Tween 80 at 37 °C under static conditions (for in vivo experiments) or with agitation (for in vitro studies) and stored in frozen stocks at minus 70 °C as described previously [21].

### 2.4. Growth Index and C_max_ of M. tuberculosis Cultures

Frozen stocks (1 × 10^5^ CFU) of mycobacteria were inoculated into 50 mL Middlebrook 7H9 broth with OADC supplement (BD, Franklin Lakes, NJ, USA) and 0.05% Tween 80 in three biological replicates incubated at 37 °C with agitation for 30 days. Every five days, aliquots of 0.1 mL were taken for CFU counting by plating the serial 10-fold dilutions in triplicates onto Middlebrook 7H11 agar (BD, Franklin Lakes, NJ, USA) enriched with OADC. To compare the growth rate among strains, we determined a growth index and C_max_ as described previously [23].

### 2.5. Antibacterial Susceptibility

Drug susceptibility tests of clinical *M. tuberculosis* strains to isoniazid (INH), rifampin (RIF), streptomycin (STR), ethambutol (EMB), amikacin (AMK), kanamycin (KAN), capreomycin (CAP), ofloxacin (OFX), and pyrazinamide (PZA) were performed by the BACTEC MGIT 960 system (BD, Sparks, MD, USA) according to the manufacturer’s instructions, and additional testing was performed using the TB test kit (SRCAMB, Obolensk, Russia) [24].

### 2.6. Whole-Genome Sequencing

Isolates were cultured on Löwenstein–Jensen (BD, Franklin Lakes, NJ, USA) slopes, and then mycobacterial genomic DNA was extracted using a DNeasy Blood & Tissue Extraction Kit (Qiagen, Hilden, Germany) following the manufacturer’s protocol. The concentration of DNA was measured with a Qubit fluorometer (ThermoFisher, Waltham, MA, USA). Samples were fragmented to 350 bp-length fragments with Covaris sonicator and were then purified with AMPure magnetic beads (ThermoFisher, Waltham, MA, USA). Paired-end libraries were prepared with an MGIEasy Universal Library Prep Set (BGI/MGI, Shenzhen, China/San Jose, CA, USA) following the instructions. Whole-genome sequencing was performed using an MGISEQ-200RS instrument (BGI/MGI, China/USA) yielding 50 bp paired-end reads. Sequence reads were deposited to the NCBI under BioProject PRJNA704837.

### 2.7. Genomic Analysis

Quality assessment of all acquired reads was performed with FastQC v.0.11.9 (http://www.bioinformatics.babraham.ac.uk/projects/fastqc/, accessed on 1 August 2021). Variant calling against *M. tuberculosis* H37Rv (NC_000962) genome was performed using the Snippy pipeline (https://github.com/tseemann/snippy, accessed on 1 August 2021). QualiMap v.2.2.2 was used to check mapping quality [25]. QC reports were aggregated with MultiQC v.1.9 [26]. A core SNP alignment was produced with snippy-core v.4.6.0 (https://github.com/tseemann/snippy, accessed on 1 August 2021); SNPs in PE/PPE/PGRS genes were masked to reduce the false positive rate. Gubbins v.2.4.1 [27] was used to filter out recombinant regions from the alignment. The resulting alignment was cleaned to include only core polymorphic sites with SNP-sites [28]. Cleaned core alignment was used to infer a phylogenetic tree via RAxML-NG v.1.0.1 [29] using the GTR + G model and autoMRE (cutoff: 0.030000) bootstrapping convergence criteria; the final tree was rooted on *M. tuberculosis* H37Rv (NC_000962). The tree was visualized with the ggtree v.2.0.2 [30] package for R v.4.0.2 [31]. WGS data for CAO and Beijing B0/W148 clusters were taken from our previous studies [11,32].

### 2.8. Mice Infection

Female C57BL/6 mice (*n* = 220) were obtained from the Scientific Center for Biomedical Technologies of the Federal Medical and Biological Agency (Andreevka, Russia). The experiment started when mice reached age 14–15 weeks and bodyweight 22–26 g. Mice were randomized into four experimental groups: control (uninfected), H37Rv, 267/47, and 120/26 (infected by corresponding *M. tuberculosis* strains). Mycobacterial cells were grown to the mid-logarithmic phase (OD_600_ = 1.0), collected by centrifugation, and washed with phosphate-buffered saline containing 0.05% Tween-80. Mice were injected into the lateral tail vein with 5 × 10^6^ CFU/mouse (in 0.1 mL of 0.9% NaCl) *M. tuberculosis* strain H37Rv and clinical strains. All animals were weighed each day after infection. Animals were observed for 150 days, the physical appearance and behavior of animals were estimated, and the daily animal weight loss and mortality were calculated. On the 14th, 30th, 60th, and 90th days postinfection (p.i.), 6 mice from each timepoint were euthanized using CO_2_ gas in each experimental group. Mouses lungs, spleen, and liver tissues were examined for mycobacterial load and pathology.

### 2.9. Mice Survival Rate and Bodyweight Dynamic

Survival rate and bodyweight dynamic of C57BL/6 mice after intravenous injection with 5 × 10^6^ CFU/mouse *M. tuberculosis* clinical strains 267/47 and 120/26 were compared to those of mice injected with a reference virulent strain, H37Rv (20 mice per strain). Additionally, as a negative control, a group of uninfected animals (*n* = 20) was used. Mouse survival was observed from the first to 150 days p.i.; bodyweight dynamic was estimated from the first to 63 days p.i.

### 2.10. Mycobacterial Load in Mice Parenchymal Organs

*M. tuberculosis* load in the lungs, spleen, and liver of C57BL/6 mice infected with the 267/47, 120/26, and H37Rv strains was calculated on the 14th, 30th, 60th, and 90th days p.i. using plating of the organ homogenates onto Middlebrook 7H11 agar with OADC supplement (BD, Franklin Lakes, NJ, USA) and growing at 37 °C for 21–28 days as described previously [21].

### 2.11. Histology of Mice Parenchymal Organs

Lung, spleen, and liver samples were fixed in 10% formalin (BioChem-NN, Nizhny Novgorod, Russia), dehydrated in ethanol and butanol gradient concentrations, embedded in paraffin, and cut on serial sections (5 µm width) using the Ultracut microtome instrument (Reichert-Jung, Bensheim, Germany). Deparaffinated sections were stained with hematoxylin/eosin (H&E) and examined with a Nikon Eclipse 80i microscope and a Nikon DS-U2 digital camera (Nikon, Tokyo, Japan) at ×4 and ×20 magnifications.

### 2.12. Semiquantitative Histologic Scoring

Images from H&E-stained slides of lung and liver tissues collected from mice on the 90th day after intravenous inoculation with the *M. tuberculosis* strains H37Rv, 267/47, and 120/26 were analyzed to generate a quantitative score. Briefly, images were taken at ×40 magnification using a Nikon Eclipse 80i microscope and a Nikon DS-U2 digital camera (Nikon, Tokyo, Japan) at multiple locations (~100 fields of view). Images were analyzed by double-blind observers. A scale from 0 to 3 was used for lung and liver slides (Table 1).

### 2.13. RNA Isolation and cDNA Synthesis

The left lungs of mice were frozen in liquid nitrogen and transferred into a microcentrifuge tube containing 1 mL of phenol/guanidine thiocyanate mix (Syntol, Moscow, Russia) and 0.2 mL of 0.1 mm zirconia/silica beads (BioSpec Products, Bartlesville, OK, USA) and homogenized using a Bead Beater instrument (Bio-Spec Products, Bartlesville, OK, USA). Total RNA was isolated by phenol–chloroform extraction as described previously [33]. All following procedures for RNA isolation were performed at 4 °C to limit RNase activity. After isolation, RNA was treated with TURBO DNase (Invitrogen, Carlsbad, CA, USA) to remove traces of genomic DNA. One µg of isolated total RNA was used for cDNA synthesis with a RevertAid RT Reverse Transcription Kit (Thermo Fisher Scientific, Waltham, MA, USA) according to the manufacturer’s protocol.

### 2.14. qPCR Detection of Cytokine mRNA in the Mice Lung

Quantitative RT-PCR was performed using qPCRmix-HS SYBR (Evrogen, Moscow, Russia) and the CFX96 Real-Time PCR system (Bio-Rad Laboratories, Hercules, CA, USA) with the following program: 95° for 20 s, 61° for 20 s, 72° for 30 s, repeat 40 times. The specific primers for revealing mice cytokine genes (Table S1) were designed using Vector NTI Advance 11.0 (Invitrogen, Carlsbad, CA, USA) and purchased from Evrogen (Moscow, Russia). Oligonucleotide secondary structures were checked with Gene Runner 6.5.52 (http://www.generunner.net/ accessed on 1 August 2021). The specificity of designed primers was verified by the NCBI primer design tool [34]. Three technical replicates per each of the six biological samples were used for statistical validity. The relative transcript levels of mice cytokine genes were calculated using the 2^−ΔΔCt^ method [35]. A heat map of changes in gene expression levels of cytokines relative to *Gapdh* and *Actb* in the lung of C57BL/6 mice on the 30th, 60th, and 90th days p.i. by the *M. tuberculosis* strains H37Rv, 267/47, and 120/26 was designed using GraphPad Prism version 8.0.1 for Windows (GraphPad Software, La Jolla, CA, USA, www.graphpad.com accessed on 1 August 2021). Gene expression levels of each cytokine at each timepoint of infection were compared to those expressed in uninfected mouse lungs.

### 2.15. Statistical Methods

Data analysis was conducted using GraphPad Prism version 8.0.1 for Windows (GraphPad Software, La Jolla, CA, USA, www.graphpad.com accessed on 1 August 2021). Survival data were analyzed using the Gehan–Breslow–Wilcoxon test. The growth index and C_max_ were compared amongst the H37Rv and clinical strains using the unpaired t-test at each timepoint. Statistical analysis between groups in the experiments on bacterial load in mice organs assessment was performed using the nonparametric Mann–Whitney test. Scores for the semiquantitative histologic scoring system were summarized for statistical comparison. To assess significance, an unpaired Welch’s test was used. A value of *p* < 0.05 was considered significant.

## 3. Results

### 3.1. Phenotyping and Genotyping Features

Two *Mycobacterium tuberculosis* clinical strains 267/47 and 120/26 were isolated from the sputum of two patients of the National Medical Research Center of Phthisiopulmonology and Infectious Diseases (Moscow, Russia) with pulmonary tuberculosis infection in 2018 (Table S2). The next-generation sequencing results and further phylogenetic analysis revealed that the strains belonged to the Beijing B0/W148 (267/47) and CAO (120/26) clusters (Figures S1 and S2). The drug susceptibility testing correlated with genetic resistance and showed that *M. tuberculosis* strain 267/47 belonged to pre-extensively drug-resistant (pre-XDR), while strain 120/26 was attributed to multidrug-resistant (MDR) *M. tuberculosis* (Table 2). In addition to the drug resistance markers, both strains had compensatory mutations in the *rpoC* gene: Gly332Ser (267/47) and Asn698Ser (120/26).

**Figure 1:**
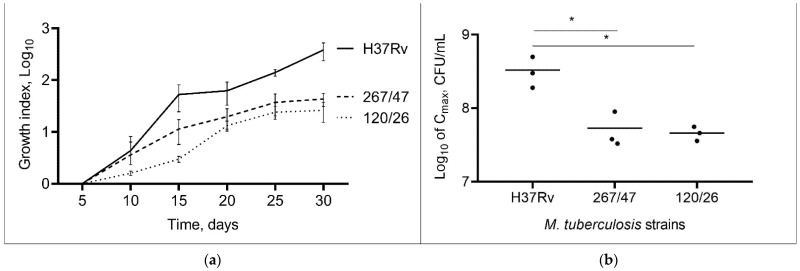
Growth dynamic of *M. tuberculosis* strains in 7H9 broth. (**a**) The growth index (calculated by dividing the colony-forming units (CFUs) at each timepoint by the CFUs at initial timepoint); (**b**) comparison of C_max_ (a maximum point on the growth curve); *—values of *p* < 0.05.

Comparison of the cultural characteristics (growth index and C_max_) of the strains showed that both clinical strains were slow-growing compared to the control H37Rv strain throughout the experimental period, *p* < 0.05. The 267/47 and 120/26 strains showed a lower C_max_ than the H37Rv strain, *p* < 0.05 (Figure 1). The C_max_ value for the 267/47 and 120/26 strains was reached on the 25th day, whilst C_max_ for the H37Rv strain was reached on the 30th day. The bacterial cell morphology of clinical strains 267/47 and 120/26 was not different from that of the same control H37Rv strain.

### 3.2. Virulence of M. tuberculosis Clinical Strains for Mice

**Figure 2:**
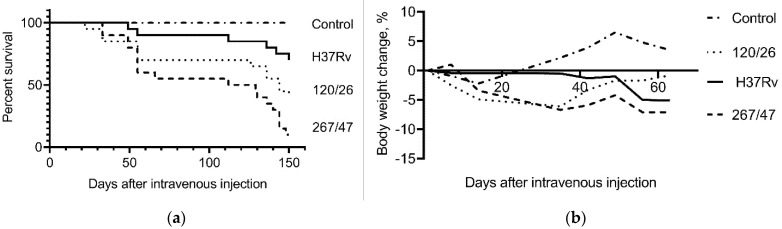
C57BL/6 mouse survival rate and bodyweight dynamic. (**a**) Survival curve of mice infected by *M. tuberculosis* strains H37Rv, 120/26, and 267/47; (**b**) bodyweight changes curves of mice infected by *M. tuberculosis* strains. Data were analyzed by the Gehan–Breslow–Wilcoxon test. The value of *p* < 0.05 was taken as statistically significant.

C57BL/6 mice infected with the 267/47, 120/26, and H37Rv strains started to die after 33, 22, and 49 days of infection, respectively. The survival rates of mice infected with 267/47, 120/26, and H37Rv strains were 10%, 40%, and 70%, respectively, on the 150th day p.i. Inoculation of mice with mycobacterial cells caused a decrease in their body weight, which significantly distinguished the experimental groups from the control group of uninfected mice (Figure 2).

### 3.3. Mycobacterial Load in Mice Parenchymal Organs

**Figure 3:**
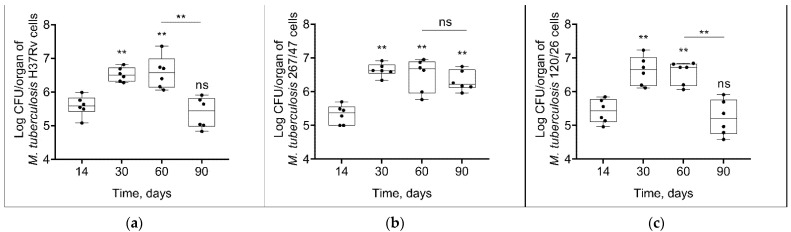
*M. tuberculosis* cell loads in the lungs of C57BL/6 mice on the 14th, 30th, 60th, and 90th days p.i.: (**a**) H37Rv; (**b**) 267/47; (**c**) 120/26. **—values of *p* ≤ 0.01; ns—not significant.

Mycobacterial load in C57BL/6 mouses lungs increased from the 14th to the 60th day p.i. in all groups of infected mice. On the 90th day p.i., the bacterial load of two strains, H37Rv and 120/26, was decreased to the initial level, in contrast with the bacterial load of 267/47 strain, which was not decreased (Figure 3).

**Figure 4:**
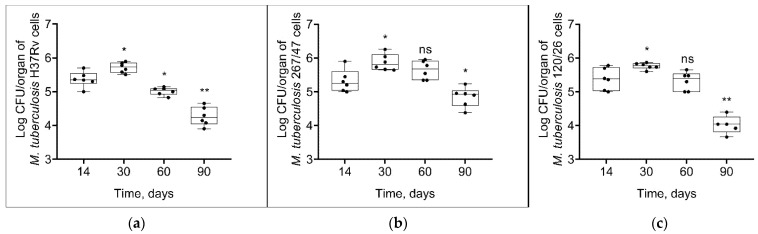
*M. tuberculosis* cell loads in the spleens of C57BL/6 mice on the 14th, 30th, 60th, and 90th days p.i.: (**a**) H37Rv; (**b**) 267/47; (**c**) 120/26. *—values of *p* ≤ 0.05; **—values of *p* ≤ 0.01; ns—not significant.

As shown in Figure 4, mycobacterial load in the spleen increased until the 30th day p.i. and then decreased until the 90th day p.i. in all groups. However, the bacterial load on the 90th day p.i. was significantly higher in the 267/47 group than in the H37Rv (*p* ≤ 0.05) and 120/26 (*p* ≤ 0.01) groups.

**Figure 5:**
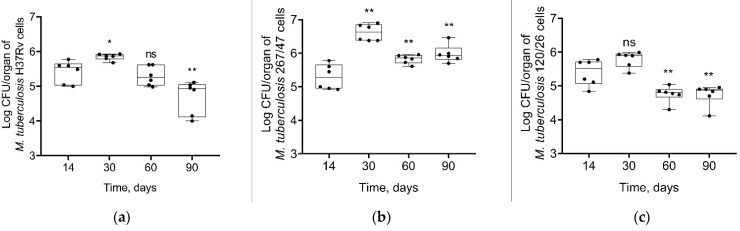
*M. tuberculosis* cell loads in the livers of C57BL/6 mice on the 14th, 30th, 60th, and 90th days p.i.: (**a**) H37Rv; (**b**) 267/47; (**c**) 120/26. *—values of *p* ≤ 0.05; **—values of *p* ≤ 0.01; ns—not significant.

Some different dynamics of bacterial load were obtained in the liver. The strain H37Rv induced a slight increase of bacterial load until 30th day p.i. followed by a decrease until the 90th day p.i. The strain 267/47 caused a significant increase in the mycobacterial load until the 30th day p.i. following by a decrease until the 60th and 90th days p.i. In contrast, bacterial loads for the strain 120/26 did not change by the 30th day p.i. and then significantly decreased until the 60th and 90th days p.i. Notably, the level of bacterial load on the 90th day p.i. induced by the strain 267/47 was significantly higher (*p* ≤ 0.01) compared to those of the strains H37Rv and 120/26 (Figure 5).

### 3.4. Histology of Mice Parenchymal Organs on 90th-Day p.i.

**Figure 6:**
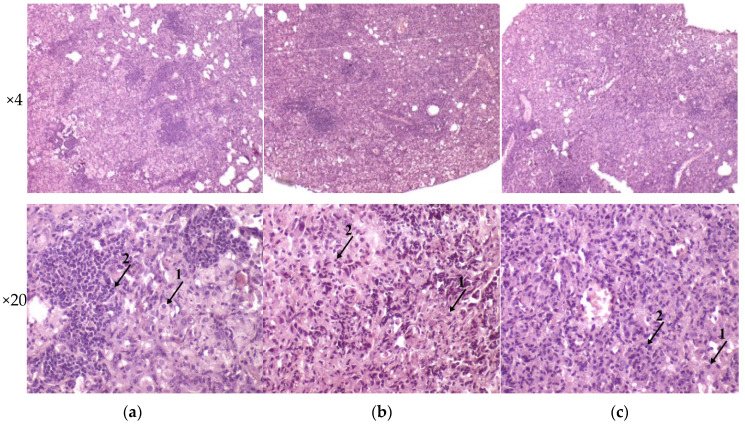

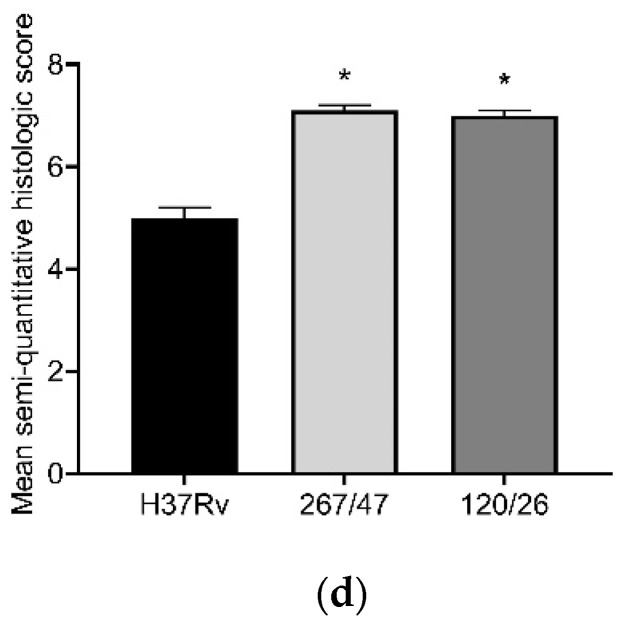
Histology of C57BL/6 mouse lungs on the 90th day after intravenous inoculation by the *M. tuberculosis* strains H37Rv (**a**), 267/47 (**b**), and 120/26 (**c**). The arrows indicate the specific mouse cells: 1—macrophages; 2—lymphocytes. Graphical representation of comparative histological semiquantitative analysis (**d**). *—values of *p* ≤ 0.05.

The histological investigation of the C57BL/6 mice infected intravenously by *M. tuberculosis* strain H37Rv showed a typical picture for TB mice models in the lungs: cellular infiltrate spread into the entire lobe of the lung. Macrophages were the major cells of the infiltrate. Compaction of the lung tissue and disappearance of the interalveolar septum was observed. In addition to macrophages, the infiltrate contained lymphocytes, which formed large clusters among the macrophage infiltrate, as well as polymorphonuclear leukocytes. Histopathology changed in the lung tissue collected from mice infected by *M. tuberculosis* strain 267/47, with some more pronounced changes compared with those generated by the strains H37Rv and 120/26. Semiquantitative histologic scoring analysis showed that histopathological changes induced by clinical strains 267/47 and 120/26 (scores 7) were significantly different from those induced by the strain H37Rv (score 5) (Figure 6).

**Figure 7:**
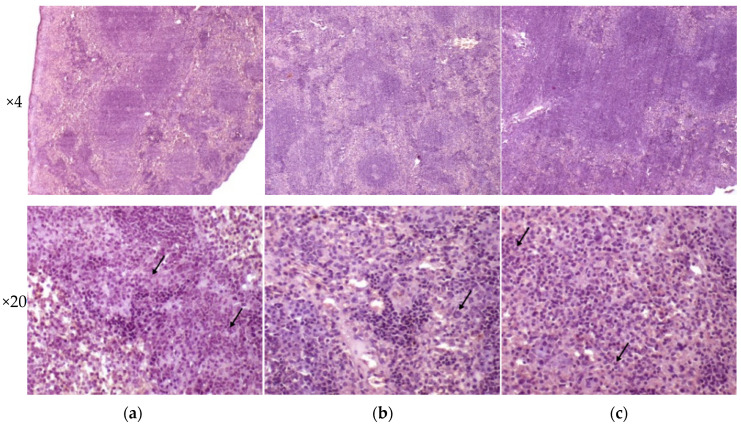
Histology of the spleen of C57BL/6 mice on the 90th day after intravenous inoculation by the *M. tuberculosis* strains H37Rv (**a**), 267/47 (**b**), and 120/26 (**c**). The arrows indicate polymorphonuclear leukocytes.

Analysis of histological pictures of the spleen revealed numerous macrophages with abundant cytoplasm in the red pulp of the spleen, many lymphocytes concentrated around macrophages, and some polymorphonuclear leukocytes. Differences were detected in the white pulp of the spleen: the strain H37Rv generated the forming of focal dense macrophage clusters with abundant cytoplasm-surrounded lymphocytes; the strain 267/47 caused a significant reduction of the white pulp in the form of separately located small lymphocyte clusters; and the strain 120/26 produced single clusters of macrophages with abundant cytoplasm. It should be noted that infection caused by the strain 267/47 resulted in the reduction of white pulp square in the spleen (Figure 7).

**Figure 8:**
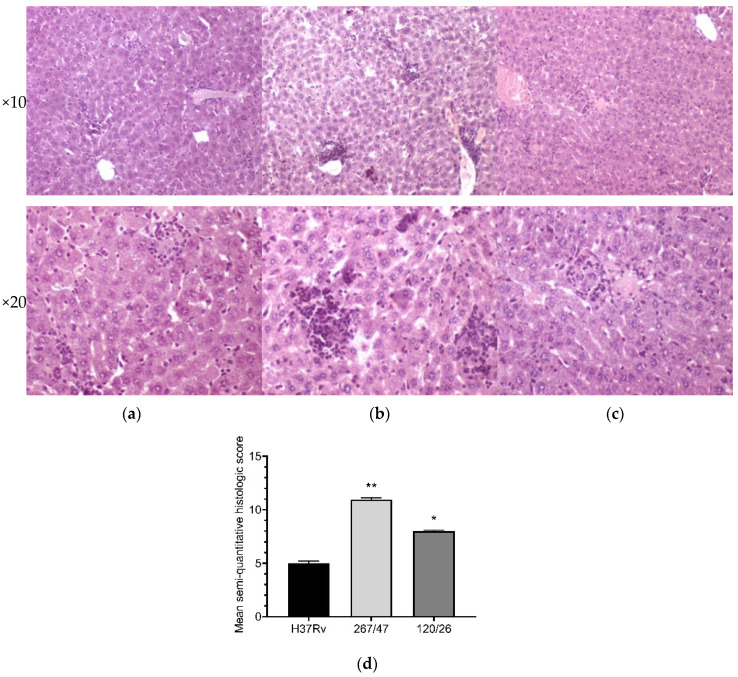
Histology of the liver of C57BL/6 mice on the 90th day after intravenous inoculation by the *M. tuberculosis* strains H37Rv (**a**), 267/47 (**b**), and 120/26 (**c**). Graphical representation of comparative histological semiquantitative analysis (**d**). **—values of *p* ≤ 0.01; *—values of *p* ≤ 0.05.

Liver tissues collected from C57BL/6 mice infected by *M. tuberculosis* strains H37Rv and 120/26 were characterized by smaller numbers of granulomas compared those induced by the strain 267/47. Granulomas in these tissue samples differed in cellular composition: the number of macrophages was significantly higher in granulomas induced by the strains 267/47 and 120/26, the number of lymphocytes was significantly higher in the granulomas generated by the strain 120/26, and polymorphonuclear leukocytes were identified in greater numbers in granulomas associated with the strain 267/47. Semiquantitative histologic scoring analysis revealed differences in total changes in liver tissues induced by the three *M. tuberculosis* strains: 267/47 (score 11), 120/26 (score 8), and H37Rv (score 5) (Figure 8).

### 3.5. Relative Cytokine mRNA Levels in the Lungs of M. tuberculosis Infected Mice

**Figure 9:**
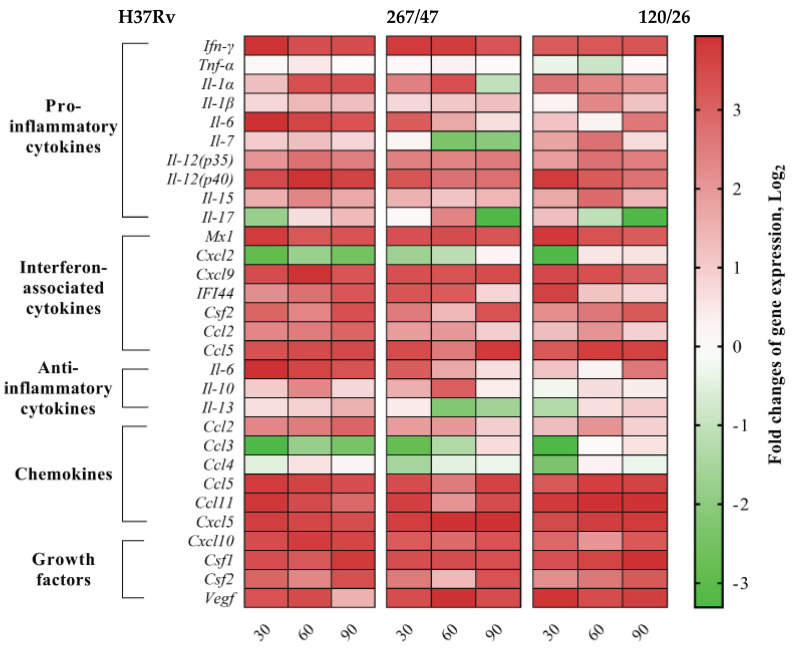
Heatmap of changes in gene expression levels of cytokine genes in the lung of C57BL/6 mice on the 30th, 60th, and 90th days p.i. caused by *M. tuberculosis* strains H37Rv, 267/47, and 120/26. Values of each cell represent log_2_ of gene expression fold changes with respect to those of uninfected mice (fold changes >1 or <−1 are significant).

We analyzed gene expression patterns of proinflammatory, interferon-associated, and anti-inflammatory cytokines, as well as chemokines and growth factors. Expression levels of proinflammatory cytokines (*Ifn-γ*, *Il-*1*β*, *Il-*12*(p*35*)*, *Il-*12*(p*40*)*, and *Il-*15), interferon-associated cytokines (*Mx*1, *Cxcl*9, *Csf*2, and *Ccl*5), chemokines (*Ccl*5, *Ccl*11, *Cxcl*5, and *Cxcl*10), and growth factors (*Csf*1, *Csf*2, and *Vegf*) were increased, and *Tnf-α* was not changed, during the observed period for all studied strains. Differences in the cytokine expression levels generated by H37Rv, 267/47, and 120/26 strains were as follows: *Il-*1*α*, *Ccl*2, *IFI-*44, *Il-*6, and *Il-*13 were consistently expressed for H37Rv from the 30th to the 90th day p.i., while *Il-*1*α*, *IFI-*44, and *Ccl*2 were decreased on the 60th or 90th days p.i. for the 267/47 and 120/26 strains. Expression of *Il-*6 was decreased on the 60th day p.i. for 267/47 and was increased on the 90th day p.i. for 120/26. *Il-*13 was downregulated from the 60th to the 90th day p.i. for 267/47 and on the 30th day p.i. for 120/26. *Il-*10 was upregulated from the 30th to the 60th day p.i. for H37Rv and 267/47 but was not changed for 120/26 during all observed periods. *Il-*7 and *Ccl*4 expression levels were not changed for H37Rv for all observed periods, but *Il-*7 was downregulated on the 60th day p.i. for 267/47 and upregulated from the 30th to the 60th day p.i. for 120/26, and *Ccl*4 was downregulated at the 30th day p.i. for both clinical strains. *Il-*17 was slightly downregulated on the 30th day p.i. and slightly upregulated on the 90th day p.i. for H37Rv, but upregulated at the 60th day p.i. and downregulated on the 90th day p.i. for 267/47, and slightly upregulated on the 30th day p.i. and downregulated from the 60th to the 90th day p.i. for 120/26. *Cxcl*2 and *Ccl*3 were consistently downregulated for H37Rv from the 30th to the 90th day p.i. and for 267/47 from the 30th to the 60th day p.i., while for 120/26, *Cxcl*2 was downregulated on the 30th day p.i. and *Ccl*3 was downregulated from the 30th to the 60th day p.i. (Figure 9).

## 4. Discussion

In this study, two *M. tuberculosis* clinical strains, 267/47 and 120/26, isolated from two patients with pulmonary TB were attributed to the Beijing B0/W148 and CAO clusters, respectively. The Beijing B0/W148 and CAO clusters are among the most studied sublineages of the Beijing genotype at the genetic level. Typical VNTR profiles and IS6110 locations have been defined for these clusters, and rapid detection systems have been implemented [36,37,38,39]. According to phylogenetic analysis and drug susceptibility tests, strains 267/47 and 120/26 demonstrated a high level of resistance similar to other members of the clusters. Moreover, the strains were closely clustered to the previously studied strains RUS_B0 [32] and Rostov [21] (Figures S1 and S2). Both *M. tuberculosis* clinical strains were slow-growing compared to the control strain H37Rv (Figure 1). In the case of strain 267/47, this is consistent with the data obtained for the strain RUS_B0 and is generally typical for resistant clinical strains [23,32]. In turn, for the strain 120/26, the opposite result compared to the strain Rostov was obtained, which was more drug-resistant and showed a faster growth rate compared to the reference strain. This may be because each strain carried different compensatory mutations in the *rpoC* gene (Asn698Ser in 120/26 vs. G332S in Rostov).

It was shown that clinical strains 267/47 and 120/26 were more virulent for C57BL/6 mice than the reference virulent strain H37Rv, with 10%, 40%, and 70% survival rates on the 150th day p.i., respectively (Figure 2). This is consistent with previously published data about the virulence of Beijing lineages B0/W148 and CAO [12,21]. It was noted previously that the higher virulence of Beijing strains was based on their ability to induce severe lung pathology rather than on increased bacterial growth in the lungs [12]. We showed that the dynamic of bacterial load in parenchymal organs (lung, spleen, and liver) of C57BL/6 mice generated by the strain 267/47 was significantly different compared with those of the strains 120/26 and H37Rv (Figure 3, Figure 4 and Figure 5). This phenomenon reflects the higher virulence potential of the strain 267/47 belonging to sublineage B0/W148. Similarly, it has been previously shown that a strain of sublineage B0/W148 was more virulent than a strain of sublineage CAO [20]. Interestingly, a similar bacterial load in C57BL/6 mice lungs, likely associated with infection generated by the strain 267/47, was described by Almeida et al. for TB caused by a hypervirulent *M. tuberculosis* strain isolated in Mozambique [40]. The histological investigation confirmed differences in the virulence of the strains under study. The strain 267/47 caused the most severe damage in parenchymal organs, while the strain 120/26 caused less pronounced damages compared to those caused by the reference strain H37Rv (Figure 6, Figure 7 and Figure 8).

Comparative analysis between groups of mice infected with *M. tuberculosis* strains of different virulence and belonging to sublineage B0/W148 and CAO, as well as the reference strain H37Rv, revealed differences in cytokine profiles (Figure 9). The expression level of cytokine *Il-*1*α* involved in innate and adaptive immune responses in mice was reduced in the lungs of C57BL/6 mice infected by the strain 267/47 on the 90th day p.i. compared to those of the strains 120/26 and H37Rv. According to published data, a deficit of IL-1α and/or IL-1β is followed by uncontrolled bacilli growth and pulmonary inflammation in mice [41]. In our study, the expression level of cytokine *Il-*7 in the lungs of C57BL/6 mice infected by the highly virulent strain 267/47 was downregulated on the 60th and 90th days p.i. On the contrary, the expression level of this cytokine in the lungs of mice infected by the strain 120/26 was upregulated on the 30th and 60th days p.i. It is known that IL-7 is involved in prolonging survival and reducing the mycobacterial load in the lung of mice [42] In the present study, the transcription level of the *Il-*17 cytokine gene in C57BL/6 mouse lungs was very differently induced by three *M. tuberculosis* strains. Clinical strains 267/47 and 120/26 were associated with downregulation of the *Il-*17 gene on the 90th and 60th–90th days p.i., respectively, in contrast with reference strain H37Rv, which was associated with downregulation on the 30th day p.i. and subsequent upregulation. These dynamics indicate different virulence mechanisms of the strains, because IL-17 is an important proinflammatory cytokine not only in early neutrophil recruitment but also in the release of cytotoxic mediators to enhance neutrophil antibacterial activity [43]. We showed that *Il-*6 gene transcription was upregulated in mouse lungs during the entire study period after intravenous injection with the strain H37Rv, in contrast to the transcription levels in mice injected with clinical strains: the strain 267/47 induced expression of this gene on the 30th day p.i.; the strain 120/26, on the 90th day p.i. It was reported previously that levels of IL-6 were higher in macrophages from TB patients than those from healthy persons [44]. Interestingly, the secretion of IL-6 by infected macrophages may contribute to the inability of IFN-γ to eradicate TB infection [45]. The transcription level of *Il-*10 was upregulated on the 60th day p.i. in the lungs of mice infected by the strains H37Rv and 267/47 but not changed in the lungs of mice infected by the strain 120/26. It was reported previously that the production of IL-10 during phagocytosis of *M. tuberculosis* cells by macrophages may be induced by bacteria and block phagosome maturation [46]. *Il-*13 was slightly upregulated on the 90th day p.i. by the strain H37Rv, while it was downregulated on the 60th–90th and 30th days p.i. by the strains 267/47 and 120/26, respectively. A previous study demonstrated the role of IL-13 in inhibiting autophagy in macrophages and suppressing intracellular survival of mycobacteria [47]. It was shown in our study that the chemokine *Ccl*4 gene was downregulated on the 30th day p.i. in mice infected with the 267/47 and 120/26 clinical strains. This is not consistent with previously published data concerning CCL4 expression detected using specific antibodies [48]. Only two chemokine genes, *Ccl*3 and *Cxcl*2, were downregulated during all experiments after TB infection caused by the strain H37Rv. Although *Ccl*3 was downregulated after infection with the clinical strains 267/47 and 120/26, the expression of this chemokine gene was restored the initial expression level by the 90th day p.i. by both strains; transcription of *Cxcl*2 gene was also downregulated after TB infection and restored to its initial level by the 90th day and 60th–90th days p.i., respectively. The role of CCL3 chemokine in TB has been proposed in the recruitment of T cells into the lung; the role of CXCL2 chemokine, recruitment of neutrophils and natural killer cells [49].

## 5. Conclusions

In this study, we confirmed that *M. tuberculosis* strains of the Beijing genotype demonstrated a different spectrum of virulence. The strain belonging to the B0/W148 sublineage (267/47) were more virulent on the C57BL/6 mice model than the strain of the CAO sublineage (120/26). To date, there have been few published studies focused on the detailed analysis of the mechanisms through which *M. tuberculosis* strains implement different levels of virulence. Therefore, the accumulation of additional knowledge in this field is very important for microbiologists, TB clinicians, and epidemiologists.

## Figures and Tables

**Table 1:** Semiquantitative histologic scoring system. Infiltration of lungs and liver tissues of mice on the 90th day after intravenous inoculation by the *Mycobacterium tuberculosis* strains.

	Score/Average Number per 100 Fields of View			
	0	1	2	3
Lungs				
Macrophages	none	≤8000	≤8500	≤9000
Lymphocytes	none	≤500	≤1000	≤2000
Polymorphonuclear leukocytes	none	≤100	≤250	≤500
Liver				
Granulomas	none	≤50	≤100	≤150
Macrophages	none	≤1000	≤2000	≤3000
Lymphocytes	none	≤500	≤1000	≤1500
Polymorphonuclear leukocytes	none	≤500	≤1000	≤1500

**Table 2:** Antibacterial resistance and drug resistance markers of *M. tuberculosis* strains 267/47 and 120/26.

Antibacterials	267/47		120/26	
	MIC, mg/L (Interpretation)	Drug Resistance Markers	MIC, mg/L (Interpretation)	Drug Resistance Markers
INH	>1 (R)	KatG Ser315Thr *inhA* (T-8A)	>1 (R)	KatG Ser315Thr
RIF	>40 (R)	RpoB Ser450Leu	>40 (R)	RpoB Ser450Leu
STR	>10 (R)	RpsL Lys43Arg	>10 (R)	RpsL Lys43Arg
EMB	>5 (R)	EmbB Gln397Arg	>5 (R)	EmbB Gly406Asp
AMK	>30 (R)	*rrs* A1401G	<30 (S)	
KAN	>30 (R)	*rrs* A1401G	<30 (S)	
CAP	>30 (R)	*rrs* A1401G	<30 (S)	
OFX	>3 (R)	GyrA Asp94Asn	<3 (S)	
PZA	>1000 (R)	*pncA* (T-11C)	<1000 (S)	

Note: INH, isoniazid; RIF, rifampin; STR, streptomycin; EMB, ethambutol; AMK, amikacin; KAN, kanamycin; CAP, capreomycin; OFX, ofloxacin; PZA, pyrazinamide; R, resistance; S, sensitivity.

## Data Availability

Whole-genome sequences were submitted into the GenBank database (Project PRJNA704837, accession numbers CP071127.1 and CP071128.1).

## Supplementary Materials

The following are available online at https://www.mdpi.com/article/10.3390/microorganisms9081792/s1, Table S1: Oligonucleotide primers designed in this study for RT-PCR with SYBR GREEN detection of C57BL/6 mouse cytokine genes, Table S2: Clinical data of the patients with pulmonary tuberculosis, Figure S1: Maximum likelihood core-SNP phylogeny of 183 CAO isolates, inferred using 6240 nonrecombinant core genome SNPs. The different colors on the branches indicate different sublineages within lineage 2, Figure S2: Maximum likelihood core-SNP phylogeny of 506 Beijing B0/W148 isolates, inferred using 7226 nonrecombinant core genome SNPs. The different colors on the branches indicate different sublineages within lineage 2.
