# Supplementary material for: MDR and Pre-XDR Clinical Mycobacterium tuberculosis Beijing Strains: Assessment of Virulence and Host Cytokine Response in Mice Infectious Model
Source: Microorganisms. 2021 Aug 23;9(8):1792. doi: 10.3390/microorganisms9081792 (PMC8400193; doi:10.3390/microorganisms9081792)
Supplement: Supplementary file 1 [file microorganisms-09-01792-s001.zip › microorganisms-1273113-supplementary/Figure Legends.pdf]

Figure S1. Maximum likelihood core-SNP phylogeny of 183 CAO isolates, inferred using 6,240 non-recombinant core genome SNPs. The different colors on the branches indicate different sublineages within lineage 2 [11]. The outside table indicates strain drug resistance. The scale bar indicates the number of nucleotide substitutions per site. The tree is rooted on M. tuberculosis H37Rv (branch length is omitted). SM - streptomycin, INH - isoniazid, RMP - rifampicin, ETH - ethionamide, EMB - ethambutol, PZA - pyrazinamide, FQ - fluoroquinolone, KAN – kanamycin, AMK – amikacin, CPR – capreomycin.

Figure S2. Maximum likelihood core-SNP phylogeny of 506 Beijing B0/W148 isolates, inferred using 7,226 non-recombinant core genome SNPs. The different colors on the branches indicate different sublineages within lineage 2 [11]. The outside table indicates strain drug resistance. The scale bar indicates the number of nucleotide substitutions per site. The tree is rooted on M. tuberculosis H37Rv (branch length is omitted). SM - streptomycin, INH - isoniazid, RMP - rifampicin, ETH - ethionamide, EMB - ethambutol, PZA - pyrazinamide, FQ - fluoroquinolone, KAN – kanamycin, AMK – amikacin, CPR – capreomycin.
