# Supplementary material for: MDR and Pre-XDR Clinical Mycobacterium tuberculosis Beijing Strains: Assessment of Virulence and Host Cytokine Response in Mice Infectious Model
Source: Microorganisms. 2021 Aug 23;9(8):1792. doi: 10.3390/microorganisms9081792 (PMC8400193; doi:10.3390/microorganisms9081792)
Supplement: Supplementary file 1 [file microorganisms-09-01792-s001.zip › microorganisms-1273113-supplementary/Table S1.pdf]

Table S1. Oligonucleotide primes designed in this study for RT-PCR with SYBR GREEN detection of mice C57BL/6 cytokine genes.

Gene name	Forward primer	Reverse primer	Size of amplicon, bp
Gapdh	GGTGAAGGTCGGTGTGAACGGATT	AAGGGGTCGTTGATGGCAACAAT	102
Actb	TGACGATATCGCTGCGCTGG	CCACGATGGAGGGGAATACA	101
Ifn-γ	TCTTCAGCAACAGCAAGGCGAAA	TCCGCTTCCTGAGGCTGGATT	147
Tnf-α	CAGAACTCCAGGCGGTGCCTAT	TGAGTGTGAGGGTCTGGGCCATA	169
Il-1α	TGTTGCTGAAGGAGTTGCCAGAAA	CCGACTTTGTTCTTTGGTGGCAAT	149
Il-1β	TCTTTGAAGTTGACGGACCCCAAA	GGACAGCCCAGGTCAAAGGTTT	65
Il-6	TTCCTCTCTGCAAGAGACTT	GGAGTGGTATCCTCTGTGAA	116
Il-7	ATGTTCCATGTTTCTTTTAGATA	CAAGGATCAGTGGAGGAATT	52
Il-10	CACTGCTATGCTGCCTGCTCTTA	CGACTGGGAAGTGGGTGCAGTTAT	93
Il-12(p35)	CACCTCAGTTTGGCCAGGGTCATT	CAGCAGGTTTCGGGACTGGCTAA	72
Il-12(p40)	TCTGCTGCTCCACAAGAAGGAAAA	CACGTGAACCGTCCGGAGTAATTT	114
Il-13	CAGTCCTGGCTCTTGCTT	GATGTTGCTCAGCTCCTCAA	110
Il-15	CTGAGGCTGGCATTCATGTCTTCA	CCAGTTGGCCTCTGTTTTAGGGA	72
Il-17	GCTACAGTGAAGGCAGCAGCGAT	TGGAGCTCACTTTTGCGCCAA	130
Mx1	GGAGAAGGTGCGGCCCTGTATT	TTCCCTGAACTCTGGTCCCCAAT	111
IFI44	CTCGTTTGACATGGCAGCAAGAAA	GCCCTCTTTCAGGTACATGCCAAT	191
Cxcl2	GGCCAGTGAACTGCGCTGTCAA	GGCGTCACACTCAAGCTCTGGAT	81
Cxcl5	GCGCCGCTGGCATTTCTGTT	TGAGGAAGGGGCAGCTTCAGCTA	66
Cxcl9	CGCTGTTCTTTTCCTCTTGGGCAT	TGGGGCAAACTGTTTGAGGTCTTT	151
Cxcl10	ATGAACCCAAGTGCTGCCGTCAT	CCCGTCATCGATATGGATGCAGTT	114
Ccl2	ACCAGCAGCAGGTGTCCCAAA	TCCTTCTTGGGGTCAGCACAG	80
Ccl3	TCTCCACCACTGCCCTTGCTGTT	TGGAATCTTCCGGCTGTAGGAGAA	122
Ccl4	CGTGTCTGCCCTCTCTCTCCTCTT	TCAGAGCCCATTGGTGCTGAGAA	75
Ccl5	ATCTCTGCAGCTGCCCTCACCAT	AGGGAGTGGTGTCCGAGCCATA	91
Ccl11	TGCAGAGCTCCACAGCGCTTCTAT	TGGGAGGTGAAGGAAGTGACCGT	58
Csf1	GCTGGCTTGGCTTGGGATGATT	TGGCACTGCCTGGGTCCTCTAT	94
Csf2	TGGAAGCATGTAGAGGCCATCAAA	AGGCGGGTCTGCACACATGTTA	131
Vegf	GGTGCACTGGACCCTGGCTTTA	GCTTCGCTGGTAGACATCCATGAA	127
