# Supplementary material for: MDR and Pre-XDR Clinical Mycobacterium tuberculosis Beijing Strains: Assessment of Virulence and Host Cytokine Response in Mice Infectious Model
Source: Microorganisms. 2021 Aug 23;9(8):1792. doi: 10.3390/microorganisms9081792 (PMC8400193; doi:10.3390/microorganisms9081792)
Supplement: Supplementary file 1 [file microorganisms-09-01792-s001.zip › microorganisms-1273113-supplementary/Table S2.pdf]

Table S2. Clinical data of the patients with pulmonary tuberculosis.

**Item**	**Patient A**	**Patient B**
Gender	Male	Male
Age, years	36	41
Bad habits	Smoking 12 years, rarely drinks alcohol	Rarely drinks alcohol
Diagnosis	Infiltrative pulmonary TB of the left lung upper lobe, being in the phase of decay, a rounded focus of infiltration 38×28 mm with a decay cavity around was revealed in the upper lobe of the left lung	Fibrous-cavernous infiltrative pulmonary TB
Surgery	Bronchial blocker introduced to the left superior lobar bronchus	Lobectomy of the left lower lobe; bronchial blocker introduced to the right superior lobar bronchus; later - to the left superior lobar bronchus
Antibacterial therapy	PZA 1.5 g, LVX 0.75 g, KAN 1.0 g, PTO 0.75 g, CS 0.5 g; replace KAN 1.0 g on PAS 10.0 g	PZA 1.5 g, PAS 9.0 g, LZD 0.6 g, BDQ 0.1 g, CS 0.5 g
Positive dynamic of therapy	Cavity reduction in the upper lobe of the left lung and partial resorption of infiltration foci; a decrease in the decay cavity and partial resorption of foci in the upper lobe of the left lung was noted after introduction of bronchial blocker	Complete and partial resorption of foci in both lungs and a significant decrease in the decay cavity in the left lung
The outcome	The patient was discharged in satisfactory condition to continue treatment at an anti-tuberculosis facility at the place of residence	The patient was discharged in satisfactory condition to continue treatment at an anti-tuberculosis facility at the place of residence
M. tuberculosis strain	120/26	267/47
Isolation date	18.04.2018	17.05.2018
Resistance phenotype	MDR	XDR
Antibacterial resistance	INH, RIF, STR, EMB	INH, RIF, STR, EMB, AMK, KAN, CAP, OFX, PZA

Note: TB, tuberculosis; PZA, pyrazinamide; LVX, levofloxacin; KAN, kanamycin; PTO, prothionamide; CS, cycloserine; LZD, linezolid; BDQ, bedaquiline; STR, streptomycin; INH, isoniazid; RIF, rifampin; EMB, ethambutol; MDR, Multidrug-resistant TB; XDR, extensively drug-resistant TB.
