# Supplementary figures and images for: MDR and Pre-XDR Clinical Mycobacterium tuberculosis Beijing Strains: Assessment of Virulence and Host Cytokine Response in Mice Infectious Model
Source: Microorganisms. 2021 Aug 23;9(8):1792. doi: 10.3390/microorganisms9081792 (PMC8400193; doi:10.3390/microorganisms9081792)

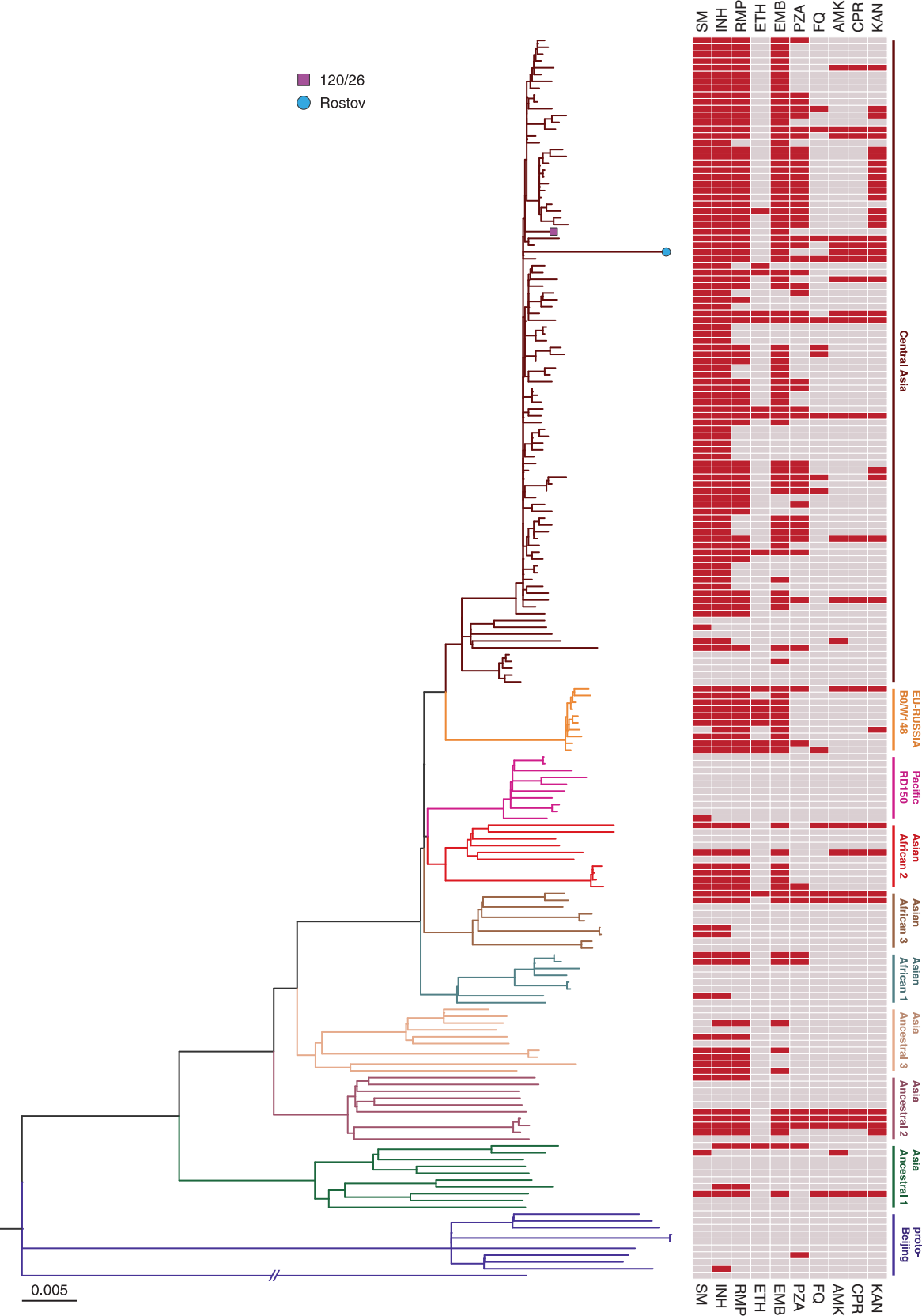

Supplement: Supplementary file 1 [file microorganisms-09-01792-s001.zip › microorganisms-1273113-supplementary/Figure S1.pdf]

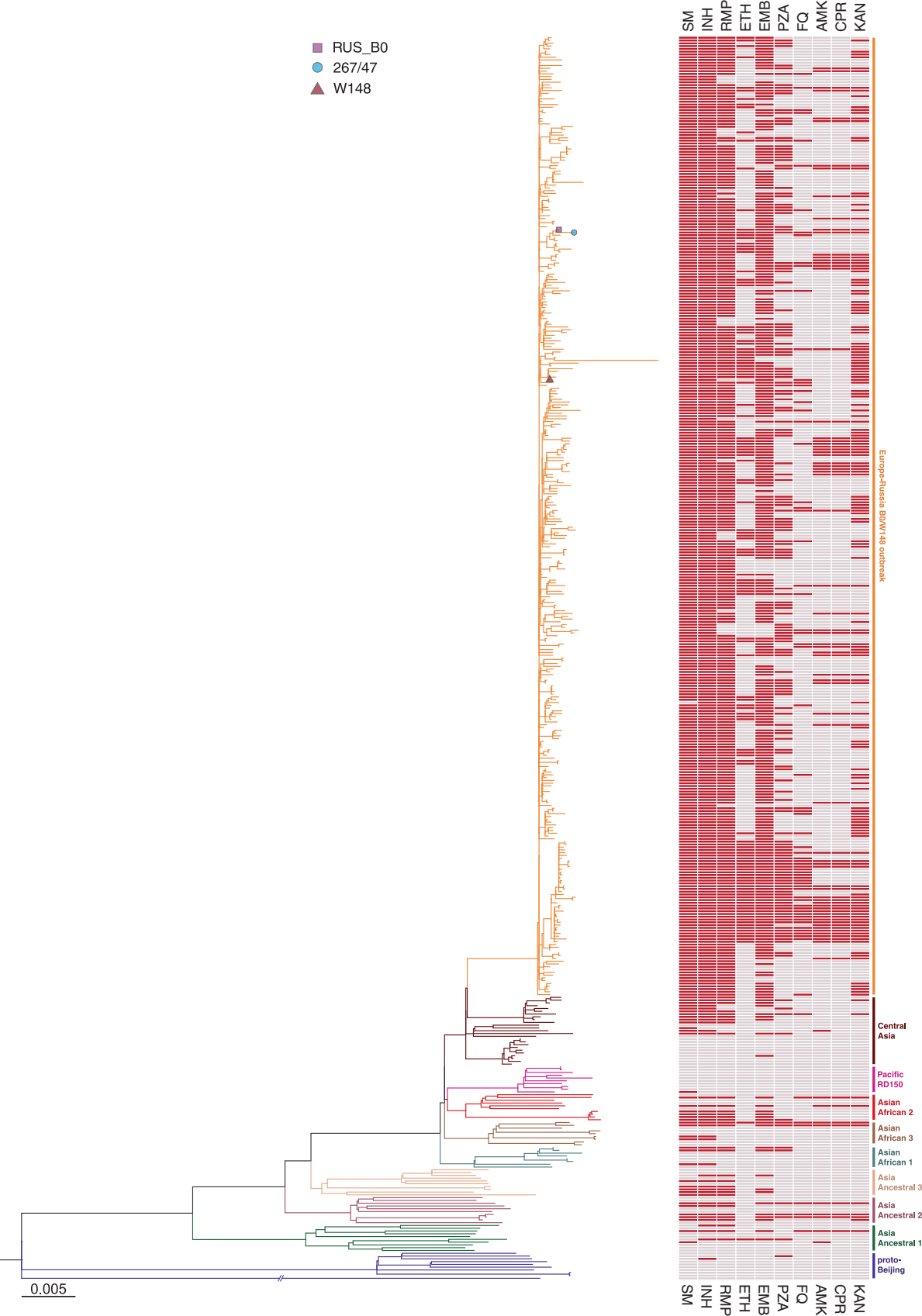

Supplement: Supplementary file 1 [file microorganisms-09-01792-s001.zip › microorganisms-1273113-supplementary/Figure S2.pdf]
